# The effect of a prenatal lifestyle intervention on glucose metabolism: results of the Norwegian Fit for Delivery randomized controlled trial

**DOI:** 10.1186/s12884-017-1340-6

**Published:** 2017-06-02

**Authors:** Linda R. Sagedal, Ingvild Vistad, Nina C. Øverby, Elling Bere, Monica K. Torstveit, Hilde Lohne-Seiler, Elisabet R. Hillesund, Are Pripp, Tore Henriksen

**Affiliations:** 1 Department of Obstetrics and Gynecology/Department of Research, Sørlandet Hospital, Postbox 416, 4604 Kristiansand, Norway; 2 Department of Public Health, Sports and Nutrition, University of Agder, Postbox 422, 4604 Kristiansand, Norway; 3 Department of Biostatistics and Epidemiology, Oslo University Hospital, Postbox 4950, Nydalen, 0424 Oslo, Norway; 4 Section of Obstetrics, Women and Children’s Division, Oslo University Hospital and University of Oslo, Postbox 4950, Nydalen, 0424 Oslo, Norway

**Keywords:** Gestational diabetes, Intervention, Lifestyle, Overweight, Obesity

## Abstract

**Background:**

The effectiveness of prenatal lifestyle intervention to prevent gestational diabetes and improve maternal glucose metabolism remains to be established. The Norwegian Fit for Delivery (NFFD) randomized, controlled trial studied the effect of a combined lifestyle intervention provided to a general population, and found significantly lower gestational weight gain among intervention participants but no improvement in obstetrical outcomes or the proportion of large infants. The aim of the present study is to examine the effect of the NFFD intervention on glucose metabolism, including an assessment of the subgroups of normal-weight and overweight/obese participants.

**Methods:**

Healthy, non-diabetic women expecting their first child, with pre-pregnancy body mass index (BMI) ≥19 kg/m^2^, age ≥ 18 years and a singleton pregnancy of ≤20 gestational-weeks were enrolled from healthcare clinics in southern Norway. Gestational weight gain was the primary endpoint. Participants (*n* = 606) were individually randomized to intervention (two dietary consultations and access to twice-weekly exercise groups) or control group (routine prenatal care). The effect of intervention on glucose metabolism was a secondary endpoint, measuring glucose (fasting and 2-h following 75-g glucose load), insulin, homeostatic assessment of insulin resistance (HOMA-IR) and leptin levels at gestational-week 30.

**Results:**

Blood samples from 557 (91.9%) women were analyzed. For the total group, intervention resulted in reduced insulin (adj. Mean diff −0.91 mU/l, *p* = 0.045) and leptin levels (adj. Mean diff -207 pmol/l, *p* = 0.021) compared to routine care, while glucose levels were unchanged. However, the effect of intervention on both fasting and 2-h glucose was modified by pre-pregnancy BMI (interaction *p* = 0.030 and *p* = 0.039, respectively). For overweight/obese women (*n* = 158), intervention was associated with increased risk of at least one glucose measurement exceeding International Association of Pregnancy and Diabetes Study Group thresholds (33.7% vs. 13.9%, adj. OR 3.89, *p* = 0.004).

**Conclusions:**

The Norwegian Fit for Delivery intervention lowered neither glucose levels nor GDM incidence, despite reductions in insulin and leptin. Prenatal combined lifestyle interventions designed for a general population may be unsuited to reduce GDM risk, particularly among overweight/obese women, who may require earlier and more targeted interventions.

**Trial registration:**

ClinicalTrials.gov ID NCT01001689, registered July 2, 2009, confirmed completed October 26, 2009 (retrospectively registered).

## Background

Maternal glucose regulation appears to be fundamentally important for fetal growth and pregnancy health. Observational studies demonstrate a linear relationship between maternal glucose levels and adverse obstetrical outcomes, particularly fetal macrosomia [1, 2], and randomized trials demonstrate that treatment of mild hyperglycemia reduces the incidence of these same outcomes [3, 4]. Gestational diabetes mellitus (GDM) is defined as hyperglycemia first detected during pregnancy, ultimately due to insufficient insulin production relative to the physiologic insulin resistance of pregnancy [5]. The level of maternal glucose that constitutes a risk for mother and fetus is much debated, and there is currently no international consensus on glucose thresholds for the diagnosis of GDM [6–8]. Effective antenatal lifestyle intervention to improve maternal glucose metabolism and reduce GDM risk is in high demand [9]. Trials published to date indicate that prenatal interventions combining diet and exercise reduce gestational weight gain but not GDM risk [10–13]. Few trials of antenatal diet and exercise have reported levels of glucose, insulin and insulin resistance [8]. These levels may give information about alterations in maternal metabolism that are not disclosed by simply reporting the incidence of GDM. Women who are overweight or obese often enter pregnancy with increased insulin resistance, and examination of glucose metabolism for this subgroup of women is therefore of particular interest [5, 14]. Leptin levels are also relevant to interventions affecting weight gain, as this adipocyte appears to play a role in glucose regulation [15].

The Norwegian Fit for Delivery (NFFD) randomized controlled trial tested the effect of a prenatal lifestyle intervention consisting of dietary counseling and supervised exercise groups on a general population including normal-weight, overweight and obese women. We have previously reported that the NFFD intervention resulted in a significant reduction in gestational weight gain (GWG) of 1.3 kg from pre-pregnancy to term but showed no significant effect of intervention on the incidence of GDM based on 2006 World Health Organization (WHO) criteria or on the proportion of large newborns [16]. The aim of the present paper is to examine the effect of intervention on levels of glucose, insulin, homeostatic assessment of insulin resistance (HOMA-IR) and leptin measured at gestational-week 30, including an assessment of intervention effect on the subgroups of normal-weight and overweight/obese women.

## Methods

**Fig. 1:**
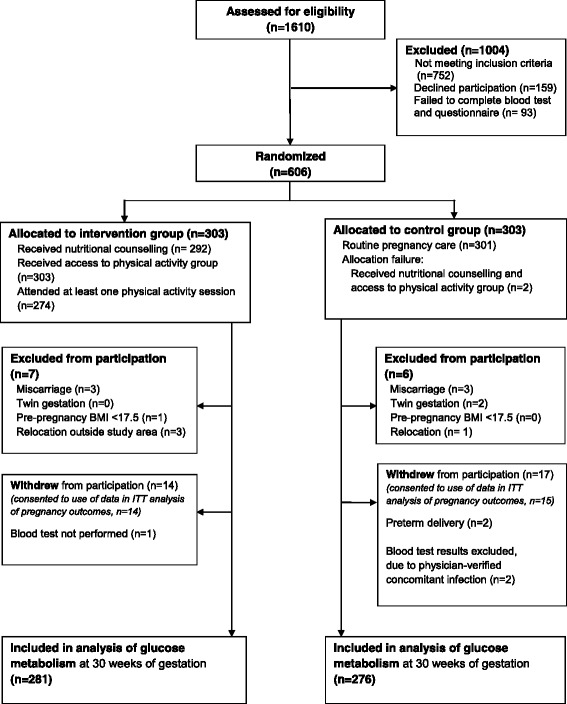
Trial profile for analysis of glucose metabolism, Norwegian Fit for Delivery trial. Blood tests were collected after fasting and two hours after glucose challenge at 30 weeks of gestation. Of 606 women randomized, 557 (91.9%) provided blood samples for analysis. An intention to treat (ITT) analysis of pregnancy outcomes included 591 women, excluding 13 from trial participation as described above and excluding two of 31 who withdrew from trial participation, due to lack of consent for use of data

NFFD is a randomized, blinded, controlled trial with two parallel groups performed in southern Norway, encompassing the cities of Kristiansand and Mandal and the more rural surrounding areas. The protocol for the trial is previously published [17]. Midwives at eight healthcare clinics enrolled participants between September 2009 and February 2013. Women were eligible if they were nulliparous, with a singleton pregnancy of ≤20 gestational weeks, had a pre-pregnancy body mass index (BMI) ≥19 kg/m^2^, were literate in Norwegian or English, and provided signed, informed consent. Exclusion criteria were pre-existing diabetes, disabilities precluding participation in a physical fitness program (based on national and international recommendations) [18], on-going substance abuse, or planned relocation outside the study area before delivery. The first 20 participants comprised a feasibility study. The protocol was modified to include a lower age limit of 18 years and to allow randomisation after initial questionnaires and blood tests were completed, in order to assure that participants were sufficiently motivated and avoid missing data. Participating clinics documented attendance of 4245 women during the inclusion period, of whom we estimate that 1610 were nulliparous (Fig. 1) [16].

### Ethics, consent and permissions

The trial was performed in accordance with the Declaration of Helsinki. The present study is a planned secondary analysis of the NFFD trial, and was included in the consent and ethical approval of the trial. The Norwegian Regional Committee for Medical Research Ethics South-East-C approved the trial and modifications (REK reference 2009/429), including specific approval for the storage and analysis of frozen serum in Research Biobank Notification no. 2594. Signed, informed consent was obtained from all participants.

### Randomisation and blinding

After receiving signed consent forms and confirming that blood tests and questionnaires were completed, a research nurse assigned participants consecutively to the intervention or control arm of the study utilizing a computer-generated list with 1:1 allocation ratio and blocks of 20. All examinations, blood test evaluations and scoring of questionnaires were performed by assessors blinded to group allocation.

### Intervention

Details of NFFD’s dietary and physical activity components and the rationale behind them are previously published [17, 19]. The dietary component was based on ten recommendations designed to increase awareness of food choices, with advice to increase intake of water, vegetables and fruit and reduce snack food consumption. There was no calorie restriction or specific limitation of fats or carbohydrates. Counselling was performed twice, by phone, with a four to six week interval. Counsellors were either experienced clinical dieticians or graduate students in public health, trained and supervised by the NFFD team. The physical activity component consisted of access to twice-weekly exercise classes at a local gym facility, led by physical therapists or students in sports science, trained and quality-controlled by the NFFD team. Attendance was recorded. Participants were encouraged to engage in 30 min of moderate-intensity physical activity on three additional days per week. Lifestyle recommendations were reinforced with booklets, access to a NFFD internet site, and invitation to one cooking class and one evening meeting with information on the NFFD trial and the value of regular exercise and healthy diet in pregnancy.

Participants in the control group received routine prenatal care following Norwegian standard: eight prenatal appointments, including one second-trimester ultrasound examination, with additional care as needed, provided free of charge. Routine care includes a booklet with advice on prenatal nutrition, physical activity and recommendations for weight gain based on current Institute of Medicine (IOM) guidelines (normal-weight: 11.5–16 kg, overweight: 7–11.5 kg, obese: 5–9 kg) [20].

### Measurements

The primary aims of the NFFD trial were to examine if intervention resulted in differences in GWG, birth weight of term infants, the proportion of term infants >4000 g, maternal fat percent at 36 gestational-weeks, and the incidence of operative deliveries. Maternal glucose levels at 30 gestational-weeks was a primary endpoint, while the proportion of women with elevated 2-h glucose tolerance tests and measurement of hormones related to glucose metabolism were secondary endpoints of the trial. Assessment of the subgroup of overweight/obese women was specified in the trial protocol.

Pre-pregnancy weight was self-reported. Participants were weighed at their healthcare clinic at inclusion, and at Sørlandet Hospital at 30 gestational-weeks (Tanita BC 418, Tokyo, Japan). Feasibility study participants reported their height; later participants were measured using a stadiometer (Seca Leicester, Hamburg, Germany). Pre-pregnancy BMI was calculated based on self-reported pre-pregnancy weight and measured height when available. Participants were weighed on admission to the delivery ward. If missing, last weight in the prenatal record was recorded with corresponding date. GWG at term was calculated for women delivering at ≥37 gestational-weeks with weight available within 2 weeks of admission.

Participants completed questionnaires at trial inclusion and at gestational-week 36, either electronically or in print. No questionnaires were completed at gestational-week 30. Diet was assessed by 43 food-frequency questions, analyzed using a pre-determined score (range 0–10, with higher score denoting healthier eating behavior). The score is previously described in detail, and has demonstrated acceptable test-retest reliability [19]. Physical activity was assessed with the International Physical Activity Questionnaire (IPAQ) short version, scored using IPAQ analysis algorithms. The IPAQ is validated in a Scandinavian population [21].

Prior to randomisation, fasting blood tests were assessed for evidence of pre-existing diabetes (defined as glucose ≥7.0 mmol/l) [22]. No participants were excluded on this basis. At gestational-week 30, plasma glucose was measured after overnight fast and again at 2-h after 75 g glucose load. All tests were performed at Sørlandet Hospital using a Cobas 6000 c501 chemistry analyzer (Roche Diagnostics). Glucose levels ≥7.0 mmol/l at fasting and/or ≥7.8 mmol/l at 2-h were classified as elevated, based on contemporary national [23] and WHO 2006 criteria [22], and participants and their primary care physicians were informed. Glucose at 2-h was missing for 12 participants (9 intervention, 3 control), primarily due to vomiting. Fasting serum samples were frozen and stored at −80 °C. Frozen samples were analyzed at Aker Hormone laboratory using a Modular E170 analyzer (Roche), batched to decrease interassay variation. Insulin was analyzed using non-competitive electrochemoluminescence immunoassay (Roche Diagnostics), with coefficient of variance of 4%. Leptin was analyzed using competitive radioimmunoassay (Millipore), with coefficient of variance of 7%. HOMA-IR was calculated as: (insulin(mU/l) x fasting glucose(mmol/l))/22.5. Leptin, insulin and HOMA-IR were missing for eight participants (3 intervention, 5 control), due to errors in freezing or transport. All missing values were considered missing completely at random. Three insulin and HOMA-IR values (1 intervention, 2 control) were excluded from analysis as outliers.

### Sample size

We predicted a 20% prevalence of newborns with birth-weight > 4000 g in the control group based on 2005 statistics from the Norwegian birth registry [24], and determined empirically that a reduction to 10% in the intervention group would be clinically relevant. We calculated that 198 women were required in each study arm to demonstrate statistical significance with a power of 80%. We also expected a 10% incidence of GDM (based on 2-h glucose ≥7.8 mmol/l) [22, 23] in the control group, and determined that a reduction to 3% in the intervention group would be clinically significant. We calculated that we would have 80% power to detect a statistically significant difference between groups with 200 participants in each arm. To allow for participant drop-out and premature deliveries and to allow for analysis of subgroups, we planned to randomize 600 participants.

### Statistics

Unadjusted comparison of intervention and control groups was performed using student t-test or chi-square test as appropriate. Difference between the randomized groups for continuous or binary variables was assessed using multiple linear or logistic regression models adjusted for age, education, income level and smoking at inclusion, pre-pregnancy BMI category and gestational age at measurement. Variables included in the adjusted analysis were chosen based on clinical relevance (pre-pregnancy BMI category and smoking) and/or measured differences between intervention and control group (gestational age at measurement) and/or measured differences between included and missing participants (age, education and income). Effect modification between randomized groups and patient characteristics on continuous outcomes was assessed by an interaction term in the multiple linear regression models. For binary outcomes, effect modification was assessed by the Breslow-Day test of homogeneity of odds ratios. No further adjustment for BMI category was performed when analysis was stratified according to pre-pregnancy BMI. *P*-values <0.05 were considered statistically significant. All tests were two-sided. We used SPSS for Windows version 21.0 for all statistical analyses.

## Results

The 606 women included in the NFFD trial were equally distributed into intervention and control groups (Fig. 1), of which 591 (97.5%) were included in a previously-published intention to treat (ITT) analysis of intervention effect on obstetrical outcomes [16]. An additional 34 women withdrew or were excluded from participation (Fig. 1) such that 557 (91.9%) women were included in the present analysis. Compared to the ITT analysis, missing participants in the intervention group (15/296, 5.1%) were younger (24.9 vs. 28.0 years, *p* = 0.005), more often without higher education (71.4% vs. 30.0%, *p* = 0.004) and reported lower income (*p* = 0.034), but had a similar distribution of occupations, pre-pregnancy BMI categories, and healthcare clinics compared with intervention participants who were tested. Missing participants in the control group (19/295, 6.4%) were not significantly different from those who were tested.

Among women in the intervention arm, 253/281 (90.0%) received both dietary consultations, 25/281 (8.9%) received one, and 3/281 (1.1%) received none. All received access to exercise classes and 267/281 (95.0%) attended at least one class. The number of classes attended prior to glucose-testing varied from 0 to 24, with median 10. The baseline characteristics of the 557 participants included in the present analysis were similar in the two groups (Table 1). Participants were predominantly white, of European descent. The majority of women in both groups were normal-weight pre-pregnancy. Five participants with pre-pregnancy BMI ≤18.5 kg/m^2^ (inclusion failures; 2 intervention and 3 control participants) were included in the normal-weight BMI category for statistical analyses. There was a similar proportion of control and intervention participants from each clinic (*p* = 0.196). Glucose-testing was performed slightly earlier in the intervention group (29.9 vs. 30.1 gestational-weeks, *p* = 0.036), such that gestational length at glucose-testing was included in adjusted analyses.

**Table 1:** Baseline characteristics of participants

	Intervention (*n* = 281)		Control (*n* = 276)	
	Mean	SD	Mean	SD
Age at trial inclusion (years)	28.0	4.2	28.0	4.5
Gestational age at inclusion (weeks)	15.4	2.7	15.6	2.5
Pre-pregnancy weight (kg)	67.5	11.9	67.3	12.4
Height (cm)	168.7	5.6	168.9	6.7
Pre-pregnancy BMI (kg/m^2^)	23.7	4.0	23.6	3.8
Inclusion weight (kg)^a^	69.8	12.2	70.1	12.6
Glucose, fasting (mmol/l)	4.43	0.38	4.45	0.40
C-Reactive Protein (mg/l)	4.38	4.26	4.36	4.04
	N	%	N	%
BMI category, pre-pregnancy				
Underweight	2	0.7	3	1.1
Normal-weight	193	68.7	201	72.8
Overweight	64	22.8	52	18.8
Obese	22	7.8	20	7.2
Education level^b^				
12 years or less	84	30.0	89	32.4
< 4 years of higher education	101	36.1	84	30.5
≥ 4 years of higher education	95	35.5	102	37.1
Occupation^c^				
Employed outside the home	230	81.9	239	86.9
Student	33	11.7	16	5.8
Unemployed	7	2.5	13	4.7
Long-term sick leave	6	2.1	4	1.4
Homemaker	5	1.8	3	1.1
Cohabitation^c^				
Husband/boyfriend/partner	274	97.5	263	95.6
Other	7	2.5	12	4.4
Household Income (NKR)^d^				
≤ 400,000	89	31.7	84	30.8
401–700,000	79	28.1	76	27.8
> 700,000	99	35.2	93	34.1
Refrained from response	14	5.0	20	7.3
Smoking status^c^				
Smoker	8	2.8	13	4.7
Non-smoker	273	97.2	262	95.3

^a^Weight at inclusion was missing for 8 (2 control and 6 intervention) participants

^b^Education information missing for 1 intervention and 1 control participant

^c^Information on occupation, cohabitation and smoking missing for 1 control participant

^d^Income information missing for 3 control participants

The intervention group showed a statistically significant reduction of GWG to term compared to controls, but GWG prior to glucose-testing was not significantly different between intervention and control groups (Table 2). There was no modification of intervention effect on GWG based on pre-pregnancy BMI category.

**Table 2:** Gestational weight gain, NFFD population

	Intervention		Control		Intervention effect					
	(*n* = 281)		(*n* = 276)		Unadjusted			Adjusted		
	Mean	SD	Mean	SD	Mean diff.	95% CI	*p*-value	Adj. Mean diff.	95% CI	*p*-value
Gestational weight gain to term (kg)										
From pre-pregnancy ^a^	14.41	6.26	15.66	5.54	−1.25	−2.28, −0.22	0.017	−1.2	−2.2, −0.2	0.021
From trial inclusion^b^	12.11	5.17	12.99	4.68	−0.89	−1.75, −0.02	0.044	−1.0	−1.8, −0.1	0.025
Gestational weight gain prior to glucose testing (kg)										
From pre-pregnancy^c^	9.22	4.67	9.86	4.37	−0.64	−0.11, 1.40	0.096	−0.52	−1.28, 0.20	0.170
From trial inclusion^d^	6.96	3.24	7.18	2.96	−0.22	−0.72, 0.30	0.407	−0.24	−0.74, 0.27	0.359

Gestational weight gain analyzed as continuous outcome variable using Student’s t-test for unadjusted comparison of intervention and control groups, and multiple regression analysis including age, smoking status, educational level and income at trial inclusion. Analysis of weight gain prior to glucose testing also included gestational length at time of measurement (analysis from pre-pregnancy) or interval between measurements (analysis from trial inclusion)

^a^Gestational weight gain to term missing for 47 participants: 31 who delivered at <37 gestational-weeks (16 intervention, 15 control) and 16 without measured weight at or within 2 weeks of delivery (9 intervention, 7 control)

^b^An additional 6 participants were without measured weight at trial inclusion (5 intervention, 1 control)

^c^Weight gain prior to glucose testing missing for 1 participant (intervention) without weight measured at glucose testing

^d^An additional 8 participants were without weight measured at inclusion (6 intervention, 2 control)

The effect of NFFD intervention on biochemical elements of glucose metabolism was assessed for the whole population and for the subgroups of normal-weight (BMI < 25, *n* = 399) and overweight/obese participants (BMI ≥ 25 kg/m^2^, *n* = 158), see Table 3.

**Table 3:** Effect of NFFD intervention on glucose regulation

	Intervention		Control		Intervention effect					
	(*n* = 281)		(*n* = 276)		Unadjusted			Adjusted		
	Mean	SD	Mean	SD	Mean diff.	95% CI	*p*-value	Adj. Mean diff.	95% CI	*p*-value
Glucose, fasting (mmol/L)										
Whole population	4.66	0.40	4.65	0.34	0.01	−0.05, 0.07	0.724	−0.00	−0.06, 0.06	0.912
BMI < 25 kg/m^2 a^	4.56	0.34	4.61	0.32	−0.05	−0.11, 0.02	0.142	−0.04	−0.10, 0.03	0.239
BMI ≥ 25 kg/m^2 b^	4.87	0.45	4.74	0.37	0.13	−0.01, 0.26	0.059	0.11	−0.02, 0.25	0.094
Glucose, 2 h (mmol/L)										
Whole population	6.07	1.34	6.08	1.16	−0.01	−0.22, 0.21	0.964	0.030	−0.18, 0.24	0.776
BMI < 25 kg/m^2^	5.84	1.15	6.03	1.09	−0.19	−0.42, 0.03	0.089	−0.16	−0.38, 0.07	0.175
BMI ≥ 25 kg/m^2^	6.59	1.57	6.20	1.32	0.39	−0.07, 0.85	0.099	0.30	−0.18, 0.77	0.217
Insulin (mU/l)										
Whole population	11.06	5.54	11.69	6.19	−0.63	−1.62, 0.36	0.210	−0.91	−1.79,-0.02	0.045
BMI < 25 kg/m^2^	9.37	4.20	10.28	5.25	−0.91	−1.86, 0.04	0.060	−0.93	−0.03, 1.88	0.056
BMI ≥ 25 kg/m^2^	14.81	6.28	15.62	6.91	−0.80	−2.89, 1.28	0.446	−0.83	−2.97, 1.31	0.468
HOMA-IR^c^										
Whole population	2.34	1.30	2.45	1.41	−0.11	−0.34, 0.11	0.332	−0.18	−0.38, 0.03	0.089
BMI < 25 kg/m^2^	1.92	0.94	2.13	1.35	−0.20	−0.41, 0.01	0.056	−0.21	−0.41, 0.01	0.056
BMI ≥ 25 kg/m^2^	3.25	1.50	3.35	1.70	−0.09	−0.60, 0.41	0.712	−0.11	−0.63, 0.42	0.692
Leptin (pmol/l)										
Whole population	2471.1	1254.1	2606.7	1215.1	−135.6	−342.7, 71.5	0.199	−207.8	−383.4, −32.1	0.021
BMI < 25 kg/m^2^	2048.0	982.9	2251.7	971.4	−203.7	−398.0, −9.3	0.040	−201.7	−395.4, −7.9	0.041
BMI ≥ 25 kg/m^2^	3415.7	1283.7	3587.9	1286.0	−172.3	−577.7, 233.1	0.403	−256.9	−662.2, 148.4	0.212

Hormone levels and HOMA-IR analyzed as continuous outcome variables using Student’s t-test for unadjusted comparison of intervention and control groups, and multiple regression analysis including age, smoking status, educational level and income at trial inclusion, and gestational length at time of testing

^a^Subpopulation with pre-pregnancy BMI < 25 kg/m^2^: intervention group *n* = 195, control group *n* = 204

^b^Subpopulation with pre-pregnancy BMI ≥ 25 kg/m^2^: intervention group *n* = 86, control group *n* = 72

^c^HOMA-IR calculated as (insulin x fasting glucose)/22.5

The NFFD intervention resulted in lower insulin levels for the intervention group vs. the control group (Table 3) and a strong trend toward lower insulin levels among normal-weight women (adj. Mean diff. -0.91 mU/l, (95%CI -1.86, 0.04), *p* = 0.056). Normal-weight women also had a trend toward reduced insulin resistance as demonstrated by lower HOMA-IR (adj. Mean diff. -0.21, (95%CI -0.041, 0.01), *p* = 0.056). Further, the intervention was associated with a significant reduction of leptin for both the whole intervention population and the subgroup of normal-weight women. For the smaller subgroup of overweight/obese women, there was no significant reduction in leptin, insulin or HOMA-IR levels as a result of intervention.

**Fig. 2:**
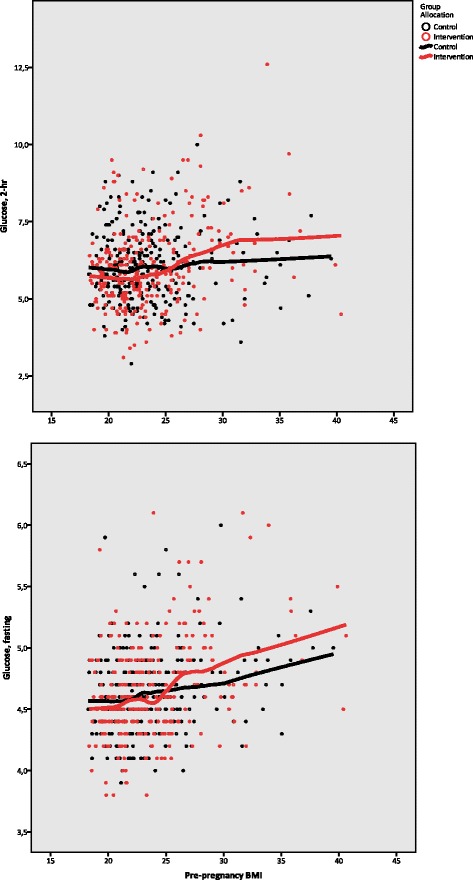
Interaction of NFFD intervention and pre-pregnancy BMI on glucose levels. Glucose measured after fasting and 2-h after 75 g glucose load

The intervention had no effect on glucose levels for the group as a whole, either fasting or 2-h after glucose challenge (Table 3). However, analysis showed a significant interaction (effect modification) between pre-pregnancy BMI category and intervention effect on glucose levels at both time points (*p* = 0.030 for fasting glucose, *p* = 0.039 for 2-h glucose), which is illustrated in Fig. 2. Among overweight/obese women, there was a trend toward slightly higher fasting glucose levels for those receiving intervention compared to controls.

As previously reported, there was no significant difference between intervention and control groups in the proportion of glucose values exceeding 2006 WHO thresholds for GDM, which are still in use in Norway. Applying proposed-revised Norwegian thresholds (fasting glucose ≥5.3 mmol/l and/or 2-h glucose ≥9.0 mmol/l), there was a trend toward a greater proportion of intervention participants with elevated glucose (8.8% vs 4.8%, adj. OR 2.01, 95%CI 0.95, 4.26, *p* = 0.069). Using thresholds recommended by the International Association of Diabetes and Pregnancy Study Groups (IADPSG) (fasting glucose ≥5.1 mmol/l and/or 2-h glucose ≥8.5 mmol/l), the intervention group had a significantly larger proportion of women with one or more elevated glucose levels compared to the control group (17.4 vs. 10.5%, adj. OR 1.8 (95%CI 1.1, 3.0) *p* = 0.029).

Assessing risk of exceeding IADPSG thresholds showed a significant modification of intervention effect by pre-pregnancy BMI category, stratified as normal-weight and overweight/obese (interaction *p* = 0.048). While the proportion of normal-weight women with glucose levels exceeding IADPSG thresholds was similar in the intervention and control groups (10.3% and 9.3% respectively, adj. OR 1.1 (95%CI 0.6, 2.2) *p* = 0.71), among overweight/obese women there was a significantly larger proportion of intervention participants with elevated glucose levels (33.7% vs. 13.9% for intervention and control group respectively, adj. OR 3.9 (95%CI 1.6, 9.7) *p* = 0.004).

Focusing on overweight/obese women in the intervention group showed that those with glucose levels exceeding IADPSG thresholds had similar GWG prior to testing, both when measured from pre-pregnancy and from trial inclusion, and attended a similar number of exercise classes (median 8 vs. nine classes, *p* = 0.283) compared to those who had lower glucose levels. There was no association between dietary score or IPAQ score at inclusion and risk of exceeding IADPSG thresholds at gestational-week 30 (*p* > 0.05), for either the intervention or control group. Glucose levels at trial inclusion were strongly associated with exceeding IADPSG thresholds at gestational-week 30 for both intervention and control groups, also after adjusting for pre-pregnancy BMI category, and age, income and educational level (*p* < 0.001). However, overweight/obese intervention participants had increased risk of exceeding IADPG thresholds also after controlling for glucose levels at inclusion in the adjusted analysis (adj. OR 4.24, *p* = 0.004).

Despite the increased proportion of intervention group women with glucose levels exceeding IADPSG thresholds, the intervention group showed no significant increase in newborn birth-weight or the proportion of large newborns, either for the group as a whole [16] or for the overweight/obese subgroup (Table 4).

**Table 4:** Neonatal outcomes for overweight/obese NFFD participants

	Intervention		Control		Intervention effect					
	(*n* = 86)		(*n* = 72)		Unadjusted			Adjusted		
	Mean	SD	Mean	SD	Mean diff.	95% CI	*p*-value	Adj. Mean diff.	95% CI	*p*-value
Gestational age at birth (weeks)	39.48	1.64	39.29	1.90	0.18	−0.37, 0.74	0.51	0.23	−0.33, 0.80	0.41
Birth weight (g)	3485	484	3466	506	19	−134, 172	0.81	−24	−138, 91	0.68
Birth weight z-score^a^	−0.16	0.82	−0.10	0.72	−0.06	−0.30, 0.18	0.63	−0.03	−0.27, 0.22	0.83
Length at birth (cm)	50.2	2.1	50.0	2.5	0.2	−0.5, 0.9	0.57	−0.2	−0.7, 0.3	0.51
Ponderal index	2.76	0.20	2.77	0.23	−0.01	−0.07, 0.06	0.86	0.01	−0.06, 0.08	0.83
	N	%	N	%	OR	95%CI	*p*-value	Adj. OR	95%CI	*p*-value
>4 kg at term	15	18.3	9	13.6	1.41	0.58, 4.48	0.44	1.30	0.46, 3.70	0.61
>4.5 kg at term	0	0	1	1.2	b	b	0.88	b	b	1.00
Birth weight > 90th percentile^a^	4	4.9	3	4.5	b	b	0.95	1.58	0.31, 8.13	0.58

Unadjusted analysis by Student t-test for continuous values and chi-square for binary outcomes. Adjusted analysis with additional variables of age, educational level, income and smoking status at inclusion, child’s sex and gestational length at delivery (sex and gestational length not included in z-score analysis, gestational length not included in analysis of gestational age at birth)

^a^z-score and percentile determined according to population-specific assessment of birth weight according to sex and gestational age [40]

^b^Analysis not performed due to small numbers

## Discussion

### Main findings

Overall, there was little beneficial effect of the NFFD lifestyle intervention on participant glucose levels, although there was a small but significant reduction of insulin and leptin levels. The intervention appeared to have divergent effect on glucose metabolism dependent on participants’ pre-pregnancy BMI status. For normal-weight women, the intervention had a weak positive effect on glucose metabolism, as evidenced by a trend (*p* < 0.1) toward reduced insulin and insulin-resistance and significantly lower leptin values, although there was no change in mean glucose levels or the proportion exceeding thresholds for GDM diagnosis. For overweight and obese women this picture was different, with a trend towards higher fasting glucose, but without any change in the other metabolic parameters. The effect of these trends on the prevalence of GDM varied depending on criteria used. When IADPSG thresholds were employed an increase in GDM was observed, whereas when using older WHO criteria there was no difference between groups.

### Interpretation

There is little information available to date on the effect of prenatal combined lifestyle interventions on glucose, insulin and leptin levels, as most trials report the effect of intervention on GDM incidence rather than biochemical parameters. The effect of NFFD intervention on the glucose metabolism of normal-weight women is consistent with the findings of Vinter et al., who reported significantly lower insulin and HOMA-IR levels at gestational-week 28–30 following lifestyle intervention, but no significant differences in glucose levels or the incidence of GDM, albeit in an exclusively overweight/obese population [25]. Among non-pregnant individuals, exercise is well documented to improve glycemic control through improved insulin sensitivity [26]. It is plausible that a combination of exercise and diet can lessen insulin resistance, without being of sufficient intensity and/or duration to change plasma glucose levels. In the NFFD intervention group, women attended a median of 10 exercise classes (9 for overweight/obese participants) over a mean of 14 weeks between inclusion and testing, while the intended attendance was twice per week. Although we lack information about total physical activity level during this period of pregnancy, it is reasonable to suppose that greater compliance might have resulted in greater intervention effect. However, varying compliance is a hallmark of clinical practice, such that the present results likely reflect the effect of providing the NFFD intervention in a general population.

The temporal sequence of changes in the biochemical and clinical parameters following lifestyle intervention in pregnancy are not well known. In the current study, the reduction of leptin found in the total NFFD intervention group may indicate that adipokines are sensitive to interventions affecting energy metabolism. Leptin is essential in energy regulation and glucose metabolism [27, 28], and is secreted by both maternal adipocytes and placental trophoblasts during pregnancy [15]. Others have found that lower mid-pregnancy leptin levels are associated with reduced insulin resistance [28]. For the child, there is evidence that maternal mid-pregnancy leptin may be an indicator of fetal growth, with lower levels associated with reduced birth weight adjusted for gestational age [29]. Adipokines such as leptin may therefore be particularly sensitive to interventions affecting energy metabolism and may precede changes in glucose levels or clinical endpoints.

The divergent effect of lifestyle intervention on glucose metabolism based on pre-pregnancy BMI has, to our knowledge, not previously been reported. However, earlier trials have shown that women with higher pre-pregnancy BMI demonstrate resistance to intervention effect. Polley et al. reported that behavioural intervention reduced excessive GWG among normal-weight women, while overweight and obese women had a trend in the opposite direction [30]. Hui et al. [31] and Phelan et al. [32] both reported that a lifestyle intervention performed in a mixed population only reduced GWG among normal-weight women, and Phelan also reported a significant treatment-by-weight interaction for gestational hypertension [32]. The BMI-modified effects of lifestyle intervention have several possible explanations, which may be synergistic. Larger women may differ from normal-weight women in their understanding of and compliance with intervention. Additionally, overweight and obese women may enter trials with a metabolic state that is less sensitive to intervention than that of normal-weight women.

For overweight/obese women participating in the NFFD trial, assignment to exercise classes may have inadvertently discouraged further leisure-time physical activity, particularly among sedentary women. Exercise routines were designed to adjust to varied fitness levels, possibly allowing larger women to limit their exertion. Larger women may also have been intimidated by classes where normal-weight women were in the majority, perhaps explaining why overweight/obese women had lower attendance than normal-weight participants. In addition, NFFD dietary recommendations were not specifically designed to reduce GDM risk and contained no advice on restriction of calories, carbohydrates or fat.

Our finding of an increased proportion of elevated glucose levels among intervention participants compared to controls was unexpected, and its significance is unclear. Reassuringly, we found no increase in large newborns among intervention participants, an outcome that is closely associated with elevated maternal glucose. Several meta-analyses have concluded that combined lifestyle interventions in pregnancy have no effect on risk of GDM, with approximately half of the included trials demonstrating a non-significant increase in risk of GDM using varied criteria [12, 13]. The recently published RADIEL study is one of only two trials, to our knowledge, to report a significant reduction in the incidence of GDM following a combined lifestyle intervention [11, 33]. While results from individual trials must be assessed with caution, comparison may provide some insight. In contrast to the NFFD trial, RADIEL participants were included pre-gestation or in early pregnancy, which may be of critical importance. There is evidence that disposition for GDM is determined prior to pregnancy, with subclinical metabolic dysfunction before conception [14, 34]. RADIEL participants were also presumably highly motivated, as they were included in the trial based on their high-risk status. In contrast, including overweight/obese women with a normal-weight population, as was done in the NFFD trial, may have undermined the potentially greater importance of lifestyle changes for this more high-risk group.

Acknowledging that the effect of intervention may vary significantly among groups and individuals is important in planning future studies. Also important, in the current analysis, the effect of intervention on GDM risk was dependent on the thresholds used. This finding illustrates the difficulty of assessing trials that employ varying criteria for GDM diagnosis, and suggests that systematic review of individual patient data (IPD analysis) may be more suitable than standard meta-analysis for exploring the effect of prenatal interventions on glucose metabolism and gestational diabetes risk.

### Strengths and limitations

The major strengths of the NFFD trial are its randomized, controlled design and the large size of the population studied, with relatively few missing values. Measured weight at inclusion and at the time of testing make it possible to accurately assess GWG and its association with metabolic findings. A major limitation of the current analysis is that, although examination of the subgroup of overweight/obese women was detailed in the trial protocol, <30% of participants were overweight/obese and the trial was not adequately powered to detect changes in smaller subgroups. Intervention effect of equivalent size may therefore be more easily detectable in the large subgroup of normal-weight women, as in the analysis of leptin. Another limitation is that due to individual randomization, women living in close proximity and attending the same clinic were often in different trial groups; it is possible that control participants were influenced by both intervention participants and clinic personnel who were informed of the purpose of the trial. While cluster randomization of clinics would have reduced such “contamination”, it would have introduced within-clinic correlations such as familial/genetic distribution, and likely required larger sample size in order to demonstrate intervention effect [35]. Due to practical and financial constraints, insulin resistance was assessed using HOMA-IR, which has shown significant correlation in pregnancy with the gold standard of the euglycemic insulin clamp [36], although an index incorporating multiple insulin measurements during glucose-testing might more accurately reflect skeletal muscle insulin resistance [37, 38]. This may be relevant in an intervention including exercise, which is proposed to lower insulin resistance at least in part through up-regulation of skeletal muscle glucose transporter protein GLUT4 [39]. Information regarding lifestyle at the time of glucose-testing is not available, limiting our assessment of the impact of diet and physical activity on biochemical results. In addition, lack of information on participants’ ethnic background and family history, both of which can affect glucose metabolism, may contribute to residual confounding. Also important, NFFD trial participants were predominantly white, European and highly educated, which may limit the external validity of results.

## Conclusions

The findings of the NFFD trial contribute to the growing evidence that GDM is difficult to prevent using combined lifestyle interventions administered during the second and third trimesters of pregnancy. Interventions aimed at a general population may miss the mark, particularly for overweight and obese women. Future research should focus on the efficacy of early intervention, preferably starting pre-pregnancy, and on methods for increasing participant motivation and compliance.

## Abbreviations

BMI: Body mass index
GDM: Gestational diabetes mellitus
GWG: Gestational weight gain
HOMA-IR: Homeostatic assessment of insulin resistance
IADPSG: International Association of Diabetes and Pregnancy Study Groups
IOM: Institute of Medicine
IPAQ: International Physical Activity Questionnaire
IPD: Individual patient data
ITT: Intention to treat
NFFD: Norwegian Fit for Delivery
WHO: World Health Organization
